# Translation of Exposure and Epidemiology for Risk Assessment: A Shifting Paradigm

**DOI:** 10.3390/ijerph17124220

**Published:** 2020-06-12

**Authors:** Judy S. LaKind, Joshua Naiman, Carol J. Burns

**Affiliations:** 1 LaKind Associates, University of Maryland School of Medicine, 106 Oakdale Avenue, Catonsville, MD 21228, USA; 2 Perelman School of Medicine, University of Pennsylvania, 3400 Civic Center Boulevard, Building 421, Philadelphia, PA 19104, USA; joshnaiman@gmail.com; 3 Burns Epidemiology Consulting, 255 W. Sunset Ct., Sanford, MI 48657, USA; cjburns.bec@gmail.com

**Keywords:** risk assessment, epidemiology, exposure, IRIS, systematic review, carcinogen

## 1. Background

Risk assessment is a well-established process used for various types of public health decision-making, such as setting chemical site clean-up levels, developing limits on exposures to chemicals in soil, water, air and food, and determining occupational exposure limits. Risk assessors are keen to use all available robust data when developing policies and regulations. This includes information from epidemiology research and the exposure assessments within that research. 

**Figure 1:**
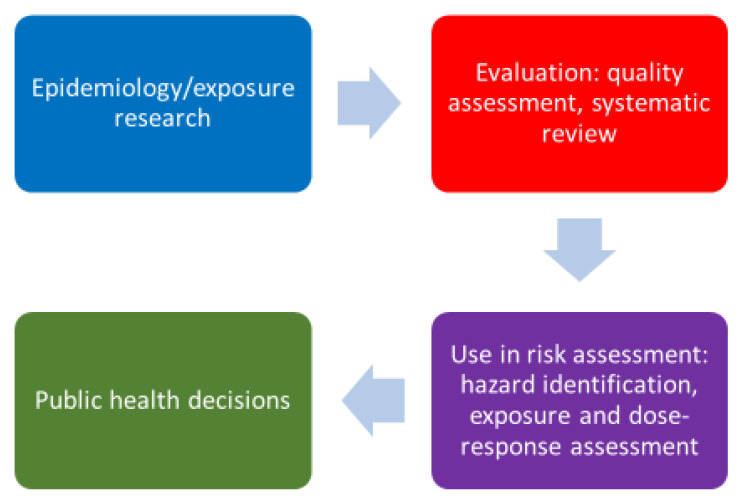
Steps from the completion of an epidemiology study to the development of an environmental health decision.

The steps from publishing an epidemiology study to seeing its use as part of the foundation for an environmental health policy decision include an assessment of the quality of the epidemiology study and a decision regarding whether the information is sufficient for use in one or more of the three risk assessment components (Figure 1). Approaches to assessing study quality have undergone rapid changes, and currently there is no single approach used consistently by agencies around the world. However, a common theme underlying these approaches is a systematic and transparent review process that examines factors such as study design, exposure assessment, outcome ascertainment methods, and control for confounding.

Investigators interested in having their research translate to public health policy will need to understand “…the mechanisms by which research findings are implemented into practice” and further will need to formulate research questions that have direct implications for decision-making [1]. This will require an understanding of the risk assessment process as well as methods regarding causal inference and study evaluation. An approach to enhancing our understanding of key aspects of risk assessment has recently been published [2] in the form of a Matrix. However, evaluation processes—which are complex and evolving—were not incorporated in the Matrix. 

Various tools have been developed to facilitate the conduct of epidemiology study quality evaluations [3,4,5,6,7,8,9,10,11,12]. The exposure assessment component—which is often described as a critical limiting factor for synthesis of results across human studies [13,14,15,16,17,18]—will likely undergo increased scrutiny by reviewers tasked with critically examining study quality. Consequently, it is possible that exposure information in epidemiology research used to support past public health decisions will no longer be viewed as sufficiently robust. We explore some examples in the following section using evaluation approaches developed by the US Environmental Protection Agency (US EPA). 

## 2. Example Exposure Assessments and Quality Evaluations

Some current research quality evaluation approaches used by the US EPA may influence—and perhaps limit—the potential translation of existing human observational research for risk assessment and decision-making. For example, chemicals are classified as “known human carcinogens” by the US EPA if there is “convincing” human evidence of a causal association between chemical exposure and cancer or if the epidemiology evidence is buttressed by other evidence, yet the criteria for determining what constitutes “convincing” evidence are evolving [19,20]. Several chemicals have been classified by the US EPA as known human carcinogens, including arsenic (inorganic), benzene, benzidine, bis(chloromethyl)ether (BCME), 1,3-butadiene, ethylene oxide, nickel subsulfide, and trichloroethylene (TCE) [21,22,23,24,25,26,27,28,29]. How would the epidemiology studies used in these assessments be viewed today using current EPA guidelines for evaluating human research? We used selected review elements from guidance developed by the US EPA Office of Research and Development for developing Integrated Risk Information System (IRIS) assessments and the EPA Office of Chemical Safety and Pollution Prevention systematic review guidance for Toxic Substances Control Act (TSCA) risk evaluations [19,20] to assess some epidemiology publications on the above chemicals; specifically:

- Does the exposure measure capture the variability in exposure among the participants, considering intensity, frequency, and duration of exposure?
- Was exposure assessed using methods known or suspected to have poor validity?
- Do the data reported address exposure scenarios (e.g., sources, pathways, routes, receptors) that are relevant to the assessment? Are the data and supporting information accessible and clearly documented? Do the data describe variability and uncertainty (quantitative and qualitative) or are the procedures, measures, methods, or models evaluated and characterized?

Overall, a review of the epidemiology publications revealed common themes regarding the exposure assessment methods. These include limited information on exposure variability, potential for exposure misclassification, and a lack of information on evidence of quality assessment and quality control. For example, the occupational studies of nickel subsulfide, benzidine and BCME equated employment with high exposure and were only able to provide analyses by exposed vs. unexposed, and/or duration of employment [30,31,32,33,34,35,36]. Thus, information on variability was not available. This issue was also noted in the US EPA’s 2011 review of TCE, in which they describe the shortcomings of reliance on employment alone due to variability in job function, year, and use of personal protection equipment [27,28]. 

With respect to the validity of exposure, a study of ethylene oxide provides a sobering example. Hornung et al. [37] used a model to predict ethylene oxide exposures but noted that the “accuracy of this model…. depends heavily upon the representativeness of the measured data. If the industrial hygienists who collected the original data used a sampling strategy weighted toward identifying overexposure problems, exposure estimates will probably be biased on the high side.” In a study of nickel subsulfide, Magnus et al. [34] noted that the classification of work categories was “very rough” due to workers moving to different sections of the plant (workers were classified according to the part of the plant where they spent the longest time). The difficulties associated with validating modeled estimates of exposure with monitored levels of 1,3-butadiene were described by butadiene cohort authors [38] who state that “…although exposure estimates are quantitative and are expressed in ppm, they are not actual measurements. The validity of the estimates is a function of the amount and accuracy of data collected at the plants, and of the assumptions used in the calculations. Thus, misclassification of exposure levels is likely.” They further note that the “exposure estimates developed for such work area groups are imprecise assessments of the exposure levels experienced by any individual worker”. Therefore, in order to meet the systematic review elements in current EPA approaches, approaches to exposure assessment with improved validity are needed.

Overall, it was difficult to evaluate study quality in many older studies since past publishing practices were marked by brevity and provided little to no information on methods for quality assurance/quality control. For example, several papers on arsenic, benzene, and benzidine included measurement data but did not contain information on sample collection methods, analytical methods, and/or quality assurance/quality control [39,40,41,42,43,44]. 

## 3. Conclusions

The importance of enhancing translation of epidemiology research for public health decision-making has been discussed in numerous publications (see, for example, Windle et al. 2019 and citations within). At the same time, approaches for evaluating the utility of epidemiology studies for use in risk assessment and for evidence-based health-protective regulations and guidance values are being developed in the US and abroad [8,19,20,45,46]. These review approaches, while differing in length and complexity, increase process transparency and share common threads for reporting, analysis and design. Thus, they can guide exposure scientists and epidemiologists who wish to design studies that will have greater use for policy decisions in the future [47]. 

In reviewing the existing epidemiology literature for several known human carcinogens, we found that while many of these studies were perhaps state-of-the-art at the time (several of the studies we reviewed dated from the 1970s and 1980s), past approaches to exposure assessment methods and reporting may now be insufficient for use by current regulatory bodies. Specifically, many of the publications reviewed were characterized by various shortcomings in the data or limited information with which to assess data quality. This could easily lead to these studies being regarded as “critically deficient” or “unacceptable”, to use the evaluation language included in the US EPA guidance documents [19,20]. 

While we are recommending that practitioners in the fields of exposure and epidemiology become familiar with evolving review approaches to enhance translation of their research, we are also aware that the numerous approaches to reviewing and evaluating studies [13,14,15,16,17,18] can result in an opacity to the process. This introduces several questions. What study elements would be required for a given study to be considered of high value? How is this being communicated to researchers, training programs and funding organizations? Will researchers have an opportunity to discuss the challenges faced by ethical, feasibility and financial constraints? While it will take time to reach a consensus on the best approach to conducting study evaluations (and there is no guarantee that consensus will be reached), still, the bar has been raised for environmental epidemiology regarding what kinds of exposure information will be considered acceptable for use in public health decisions. A better understanding of the evolving systematic review approaches and the criteria by which research will be evaluated can lead to more science-based policies derived from epidemiology studies.
